# The examination of physical function and cognitive outcomes in middle-to-older high-risk adults: an unsupervised clustering method

**DOI:** 10.3389/fpubh.2025.1351658

**Published:** 2025-05-20

**Authors:** Joshua L. Gills, Megan D. Jones, Anthony Campitelli, Sally Paulson, Cody Diehl, Charles Rodgers, Erica Madero, Jennifer R. Myers, Kelsey Bryk, Omonigho Michael Bubu, Jordan M. Glenn, Michelle Gray

**Affiliations:** ^1^Department of Psychiatry, Aging Research in Sleep Equity & Dementia Prevention Program, Institute of Excellence in Health Equity, New York University Grossman School of Medicine, New York, NY, United States; ^2^Department of Population Health, New York University Grossman School of Medicine, New York, NY, United States; ^3^Exercise Science Research Center, University of Arkansas, Fayetteville, AR, United States; ^4^Health and Human Performance Department, College of Idaho, Caldwell, ID, United States; ^5^St. Elizabeth Healthcare, Edgewood, KY, United States; ^6^University of Houston, Houston, TX, United States; ^7^University of Utah Health, Salt Lake County, UT, United States; ^8^Neurotrack Technologies, Inc., Redwoods City, CA, United States; ^9^Department of Neurology, NYU Alzheimer’s Disease Research Center, NYU Grossman School of Medicine, New York, NY, United States

**Keywords:** exercise, cognition, cardiovascular, muscles, aging, physical function

## Abstract

Alzheimer’s disease rates are expected to triple by 2050. Early detection and specific mitigation efforts are warranted to blunt the alarming increase. Physical function index (PFI) declines with age; additionally, higher PFI is associated with better cognitive functioning in middle-to-older age individuals. However, most studies utilize one domain of PFI to examine associations with cognition. Therefore, using clustering methods, the purpose of this investigation was to determine if high-risk individuals with higher PFI have better cognitive outcomes compared to individuals with lower PFI. Participants (*n* = 215; 73.1% female; 45–75 years) completed a body mass scan, venous blood draw, 7 PFI tasks, and 7 cognitive tests. A *k*-means cluster analysis was utilized to identify PFI cluster for participants, one-way ANCOVAs were used to assess differences in cognition among clusters. Cluster 1 (C1; *n* = 29) was characterized as the highest strength/power, faster dual-task walking time, and higher aerobic capacity, Cluster 3 (C3; *n* = 113) had the lowest values between PFI groups, Cluster 2 (C3; *n* = 74) was in-between C1 and C3. Individuals in C1 had significantly higher global cognitive, visuospatial scores, digital executive functioning and associative learning compared to individuals in C3 (*p* < 0.05). Individuals in C1 and C2 had significantly higher values on orientation task and figure recall than individuals in C3 (*p* < 0.05). The results from this current study demonstrate that individuals with higher combined PFI output have higher global cognitive scores than individuals with lower combined PFI output. Examining PFI variables together may be a valuable tool when assessing cognition among cognitively at-risk individuals.

## Introduction

The rates of Alzheimer’s disease (AD) are steadily rising as the aging population increases (1, 2). As of 2018, approximately 5.7 million Americans were diagnosed with AD, with an additional person being diagnosed every 65 s (1). Furthermore, AD is the sixth leading cause of death and responsible for more than 80,000 deaths annually in the United States (US) (2). The rise in rates of AD coupled with the increasing number of older adults is a major contributor to increased long-term healthcare costs in the US. As a result, early detection and tracking brain health in the aging population is now more important than ever. The earlier cognitive decline is detected, the more likely clinicians can implement programs to improve modifiable risk factors leading to improved cognitive outcomes.

Various risk factors such as age, sex, genetics, and education often effect cognition but are non-modifiable (age, sex, or genetics) or may not be easily accessible in older adults (education) (1, 2). One modifiable risk factor is exercise including aerobic fitness. The improvement and maintenance of aerobic fitness in an high-risk population offset decline in cognitive scores (memory and processing), increases cerebral blood flow, and maintains brain volume (3, 4). Another mode of exercise, muscular fitness (strength and power), improves brain function through the upregulation of brain derived neurotropic factor through induced Insulin-like Growth Factor 1 levels; higher muscular fitness is also associated with higher processing speed and executive function (5, 6). Lastly, gait speed and dual-task gait speed have exhibited strong relationships with global cognitive scores and declarative memory (7–9). The combination of these three components, termed physical function, may tell a greater story about an individual’s cognitive function than one component alone.

Furthermore, numerous interventions increasing physical activity and exercise have demonstrated improved cognitive outcomes in middle to older-aged adults (4, 10, 11). However, it remains to be seen whether individuals with high physical function, despite multiple cognitive risk factors, demonstrate higher global cognitive functioning than individuals with lower physical function. Physical function deficits later in life are related to cognitive decline (12–16). But, physical function variables, such as aerobic capacity, gait speed, and muscular fitness, combined, have not been used to evaluate cognition in mid-to-late life high-risk adults. High Risk, in this instance, is quantified by the Australian National University Alzheimer’s Disease Risk Index in which an individual with two or more risk factors for cognitive decline (Table 1). Understanding whether combined physical function output can delineate cognitive ability in high-risk individuals before clinical decline occurs may provide health care professionals with a time-efficient and cost-effective method of evaluating cognition.

**Table 1:** Inclusion and exclusion criteria.

Inclusion	Exclusion
Age 45–75 BMI 18.5–39.9 kg/m^2^ Fluent in English (written and spoken) Subjective cognitive decline with worry A minimum of 2 risk factors for AD on ANU-ADRI: High school education or less BMI 25–39.9 kg/m^2^ (overweight, obese class I or II) History of diabetes History of hypertension History of high cholesterol History of smoking Maximum of 1 protective factor for AD on ANU-ADRI: High level of physical activity High fish consumption High level of cognitive engagement Ability to send and receive text messages Access to a smartphone or tablet with a screen-side camera and reliable internet connection Ability to participate in light to moderate physical activity Willing to authorize release of medical records	Physician diagnosis of mental health condition (e.g., eating disorder, alcohol/substance use, schizophrenia, etc.) neurologic conditions (e.g., epilepsy, recent stroke, multiple sclerosis, Parkinson’s disease, brain tumor, or severe traumatic brain injury) dementia, probable dementia, or mild cognitive impairment other significant health condition (e.g., congestive heart failure, chronic obstructive pulmonary disease, coronary artery disease, renal failure, chronic kidney disease, pulmonary hypertension) Recent cardiovascular event or treatment for cancer (within the last year); on dialysis; or on active organ transplant list Visual problems that prevent viewing screen at a normal distance (e.g., legal blindness, detached retina, occlusive cataracts) History of learning disability Currently participating in a cognitive training coaching program or other lifestyle change program (e.g., diabetes prevention program) Currently pregnant or planning on becoming pregnant in the next 2 years

Therefore, the purpose of this investigation was to understand if high-risk individuals with higher Physical Function or a higher physical function index demonstrate better cognitive outcomes than individuals with lower physical function index. We hypothesize high-risk individuals with higher Physical function index will display higher cognitive domain scores compared to high-risk individuals with lower Physical function index.

## Methods

### Study design and setting

This was an exploratory, cross-sectional design where participants were recruited on a rolling basis (~6 months) from the community in Northwest Arkansas (suburban/rural) and surrounding rural areas. Data was collected from January 2021 through June 2021. Data was collected in the Exercise Science Research Center in multiple rooms dependent on examination (i.e., quiet rooms for cognitive testing, larger spaces for functional assessments). All experimental protocols were approved by the University of Arkansas’ Institutional Review Board.

### Participants

We tested 215 adults (male and female, ages 45–75 years) at increased risk for AD. During the visit, participants signed an informed consent approved by the University of Arkansas’ Institutional Review Board, provided a blood sample, underwent a body composition analysis, and completed a series of cognitive and physical measures. Inclusion and exclusion criteria are in Table 1. High-risk for cognitive decline was determined by the Australian National University Alzheimer’s Disease Risk Index (ANU-ADRI) (17). If participants had two or more risk factors for cognitive decline, they were considered high-risk and included in this investigation (Table 1). Participants were screened via survey, a follow-up visit was completed if researchers were not sure if the participant qualified. During this visit a blood pressure, body mass index (BMI), and cholesterol/glucose screening were completed.

### Physical function index, sociodemographic, and anthropometric measures

#### Demographics and anthropometric assessments

Assessments included age, sex, education, height, weight, and body composition (fat free mass, fat mass, bone mineral density), blood pressure, fasting cholesterol and glucose. Height was measured with a standing stadiometer (Seca; Hamburg, Deutschland). During this assessment, participants were asked to remove their shoes and stand up as straight as possible. Height was recorded to the nearest 0.1 cm. Weight was measured with a balance-beam scale (Detecto, Webb City, MO). Participants removed their shoes, any heavy clothing (sweaters, jackets, or coats), and empty their pockets. Weight was measured to the nearest 0.1 kg. Body composition was measured through a dual energy X-ray absorptiometry scan (General Electric Company, Madison, WI). To assess clinical measurements such as cholesterol and fasting glucose, a venous blood draw was taken at the beginning of the visit (participants were instructed to fast for at least 3 h) and examined through Cholestech (Stat-technolgies, Golden Valley, MN). After 5-min of quiet rest, SBP and DBP blood pressure was assessed 3 times in a seated position using the automatic Mircolife Blood Pressure Monitor (Microlife USA, Inc., Clearwater, FL) and averaged.

#### Alzheimer’s disease risk

The ANU-ADRI is a self-report survey instrument assessing ADRD risk by quantifying several positive and negative risk factors and applying statistically-derived weightings (17). Protective (negative risk) factors included are social engagement, cognitive activity, physical activity level, non-fried fish and seafood consumption, and alcohol consumption (if less than 2 drinks per day). Risk (positive risk) factors included in the ANU-ADRI are diabetes/dysregulated blood glucose status, depression status, obesity, history of traumatic brain injury, history of smoking, high cholesterol, high alcohol consumption (3 or more drinks per day), exposure to pesticides, as well as known demographic risk factors such as sex, age, and level of education (17). The ANU-ADRI is a valid (18) and reliable (19) measure of ADRD risk.

#### Six-minute walking distance test (6MWDT)

The 6MWDT is a field measure of aerobic capacity, with a test–retest reliability (ICC = 0.96), a minimal clinical important difference (MCID) of 20.0 meters, and adequate stability over time (20). From a standing start, participants were instructed to walk continuously for 6 min back and forth between a 25-meter distance (around a cone on both ends), labeled by study staff, at the fastest pace they felt they could maintain throughout the duration of the examination. Participants were told their goal was to cover as much ground as possible in 6 min. Participants were allowed to stop and stand or sit in a chair if needed and were instructed to walk again when they felt able. Distance walked was recorded at the end of the test to the nearest 0.1 m.

#### Hand-grip strength

Hand-grip testing was used as a measure of isometric strength as it is correlated with overall strength and functional independence (21–23). All measurements were administered by a trained technician and measured in kilograms using a handheld dynamometer (Creative Health Products, Inc., Ann Arbor, MI). All measurements were performed on each hand with the participant standing, arm down at the side, wrist in neutral position, and interphalangeal joint of the index finger maintained at 90 degrees. Participants were instructed to maximally squeeze the handle for 3–5 s with standard encouragement provided. The test was administered three times with 60s rest between attempts. The average between the three trials was used for each hand. This test has shown test–retest reliability with an ICC of 0.95, MCID of 6.5 kg (19.5%) (15).

#### Lower-body muscular power

Sit-to-stand (STS) power was measured using the Tendo Weightlifting Analyzer (Trencin, Slovac Republic). The Tendo was attached to the side of each participant by securing a belt around the participant’s waist. To ensure consistency, the Tendo was placed on the participant’s left side, with the Kevlar string positioned in the sagittal plane, when the participant is in the standing position. From a seated position, with the arms placed across opposing shoulders, the participant was instructed to stand as quickly as possible before slowly returning to the initial seated position (outcome measures only consider the egress component of the STS). As the participant stood as quickly as possible, the Tendo’s Kevlar string was pulled and power (W), partial power (W), peak power output (W), velocity (m/s) and peak velocity (m/s), and peak force (N) for each stand was recorded. Five repetitions were recorded with a 60s rest between each repetition. Average power was calculated as the mean power generated among all 5 repetitions and peak power was determined as the average among the 5 peak power repetitions recorded. Average partial power (W) was calculated by the average of all 5 repetitions and used in the analyses. Average velocity (m/s) was calculated as the mean velocity generated among all 5 repetitions and peak velocity was determined as the average among the 5 peak velocity repetitions. Peak force was measured in Newtons, it was the average among of all 5 attempts. These measures were validated for use in middle-age and older adults (24, 25).

#### 10-meter dual-task

The dual-task walking assessment evaluates physical function, attention and executive function (26, 27). This assessment has been described in detail elsewhere (8). Dual-task assessments vary in protocol, but for the purposes of this study, participants were instructed to walk 20 meters at their usual speed while time was recorded by the researcher. There was a 5-meter distance before and after the 10-meter distance to account for acceleration and deceleration (8). For the next part of the assessment, participants were instructed to walk as quickly and safely as possible without running. These two assessments were used as the baseline tests (the two baseline tests were averaged and included in the analyses). For the dual-task conditions, participants were instructed to perform the same walking conditions and simultaneously perform serial subtractions (28). A random 3-digit number between 199 and 999 was selected and participants were instructed to subtract three from each number while performing each walking condition. Four testing trials were completed, two at usual speed (dual-task habitual speed--DTHS) and two at their maximal speed (dual-task maximal speed--DTMS). Dual-task decrement was calculated as the difference between the walk trial while performing serial subtractions and the trial without subtraction. The walking speed trials (DTHS and DTMS) were averaged separately and used for all analyses.

#### 4-meter dual-task walk

Dual-task assessments vary in protocol, but for the purposes of this study, participants were instructed to walk 4 meters at their usual speed while time was recorded by the researcher. For the next part of the assessment, participants were instructed to walk as quickly and safely as possible without running. These two assessments were used as the baseline tests (the two baseline tests were averaged and included in the analyses). For the dual-task conditions, participants were instructed to perform the same walking conditions and simultaneously perform serial subtractions (28). The same protocol was used as in the dual-task section above. This test has test–retest reliability (ICC: 0.92, CI95%: 0.85–0.96) (29).

### Cognitive measures

#### Image pairs

Image pairs is an eye tracking task that measures visual recognition memory and learning (7, 30, 31). The visual paired comparison portion of the test measured the participant’s ability to recognize images they have already viewed during a familiarization phase. The paired recognition trial portion of the test measured the participant’s ability to learn and identify image pairs they have been tasked with learning. The image pairs exam is accurate with test–retest reliability (7, 30).

#### Symbol Match

Symbol Match is a processing speed and executive functioning task that utilizes a paired verification or rejection paradigm (forced choice) (32, 33). Participants were instructed to determine whether two symbols are equal or unequal utilizing a legend with nine number/symbol pairs. At the conclusion of the task, a brief implicit learning trial was administered without the legend present.

#### Arrow Match

The Arrow Match test is a measure of attention and processing speed (33). Participants were shown five arrows in the middle of the screen and were instructed to identify the direction of the middle arrow. The arrows pointed in either the same direction or in the opposite direction from the other arrows. Participants were presented with 32 trials and scores were reported as the number of correct responses relative to the time elapsed during all trials.

#### Item Price

Item Price is a brief visual paired associates paradigm (33). This task required participants to learn eight food/price pairs and discriminate between target and foil (items previously present but not paired) pairs during a recognition trial. All items belonged to the same semantic category (fruits, vegetables, etc.) and were presented in pseudorandom order using a blocking scheme.

#### Path Points

Executive function was assessed using the Path Points test. Similar to the paper-pencil Trail Making Test Part B (34), Path Points is a digital version where participants connected a series of alternating numbers and letters from 1-A to 7-G. Scores were reported as the amount of time required to complete the 14 responses. Only correct responses are allowed.

#### Light Reaction

Reaction time and inhibition was assessed with the Light Reaction test (33). Participants were presented with either a positive stimulus (green light) or negative stimulus (red light). If the positive stimulus appears, they were tasked with pressing a button. If the negative stimulus appears, they were tasked with refraining from pressing the button. Average response time for reacting to the positive stimulus (green light) was recorded.

#### Repeatable battery for neuropsychological status (RBANS)

Each participant was individually administered the RBANS assessment (RBANS; Form A). The RBANS assessment was completed on an iPad along with paper and pencil. The RBANS assessment construction is explained in detail elsewhere (35). Briefly, RBANS is made up of 12 subtests that are used to calculate five index scores and a total score. Test catalogues included: Immediate memory (list learning and story memory tasks), visuospatial/constructional (comprised of figure copy and orientation tasks), language (picture naming and semantic fluency tasks), attention (digit span and coding tasks), and delayed memory (list recall, story recall, figure recall, and list recognition tasks). Each index score falls within an age-adjusted score (35). The index scores were combined to produce a total score, which is a summary score of the participant’s performance on RBANS. The RBANS test took approximately 30 min to administer and complete. Previous research showed RBANS is significantly correlated with more extensive exams such as the Wechsler Adult Intelligence Scale III (r = 0.79, df = 148, *p* < 0.001) and the Wechsler Memory Scale III (r = 0.36, df = 144, *p* < 0.0001), it also has ICC score of 0.77 to demonstrate test–retest reliability (36, 37).

### Covariates

The following variables are known to affect cognition with potential confounding effects: age, sex, education and Apolipoprotein E (ApoE) (1). Age was characterized as a continuous variable (all participants were between 45 through 75 years of age). Sex was measured using dichotomous indicators for *male* and *female*. Education was measured using dichotomous indicators for *less than high school*, *high school degree*, *some college degree*, and *some education beyond college*. To account for the ApoE gene, a venous blood draw was taken at the beginning of the visit (participants were instructed to fast for at least 3 h). Samples were stored at −80^0^\u00B0C until analysis. Genetic testing was completed through real time polymerase chain reaction (PCR) to account for ApoE ε4, an additional risk factor for AD, by a third-party laboratory. For SNP Genotyping: DNA was extracted from participant’s whole blood. ApoE alleles were observed through SNPs rs7412 and rs429358 that were genotyped using real time PCR for 215 participants by a third party (CD Genomics, Inc., Shirley, NY). *e_2_* genotype was determined by T for SNP rs7412 and T on SNP rs429358, *e_3_* genotype was determined by C for SNP rs7412 and T on SNP rs429358, *e_4_* genotype was determined by C for SNP rs7412 and C on SNP rs429358 (38). ApoE gene carriers (*e_4_* genotype) and non-carriers (*e_2-3_* genotype) were dichotomized in the dataset.

### Statistical analysis

All data were inspected to identify missing items and outliers. Conclusions were formed that are robust to different missing-data mechanisms (39). All data were subjected to quality control checks prior to proposed statistical analysis. Assumptions for ANCOVA were assessed including heterogeneity, independence, and normality. Demographic and efficacy data were summarized by mean ± SD and categorical variables were presented as percentages. Hypothesis testing was carried out at the 5% (2-sided) significance level unless otherwise specified, and *p*-values were rounded to three decimal places. SPSS (version 26) was used for descriptive calculations and ANCOVA comparisons.

A hierarchical cluster analysis (HCA; Ward’s method based on squared Euclidean distances) was utilized to identify patterns based Physical function index levels among participants. We then categorized the participants’ data with K-means, an iterative distance-based clustering method. Briefly (40), a specific number of clusters (*k*), three based on patterns in the HCA, were selected in advance. Followed by the *k* points randomly selected as centers. All data cases were allocated to the closest center based on Euclidean distance. The cluster centers were calculated as the mean of all cases belonging to each cluster. This process was repeated until the same points were assigned to the same cluster in consecutive iterations, a total of 7 iterations were completed. These physical function index variables were included in both cluster analyses: STS variables (average power and velocity, peak power and velocity, average partial power, and peak force), handgrip, (6MWDT, 4-meter fast walking, 10-meter fast walking, 4-meter DTHS, 4-meter DTMS, 10-meter DTHS, 10-meter DTMS). One-way ANCOVAs determined the differences in cognitive domain scores and individual cognitive tests between each physical function index cluster. A Bonferroni post-hoc test was used to assess multiple comparisons between groups if ANCOVA identified significant differences. Age (not controlled for in digital cognitive tasks because the scores were age-adjusted), sex, education, APOE, and ANU-ADRI total score were co-variates in each model. To avoid bias, multiple testers and scorers were used to administer and score data. Additionally, a third-party biostatistician validated the results obtained from this study.

#### Sample size and power calculation

Based on power analysis in G power to document that we have a sufficient sample size, we anticipated exceeding 95% power to detect meaningful associations between proposed variables to delineate differences between groups with age, sex, education and ApoE used as covariates. Additionally, partial eta-squared (η^2^_p_) was utilized to demonstrate effect size. The following criteria for η^2^_p_ was used to explain the practical significance of the findings: small (0.01), moderate (0.06), and large (0.14).

## Results

### Demographic information

Of the 215 participants who completed the study, the mean age was 59.7 ± 14.1 years, 73.1% female (158 participants), and 97% Caucasian Americans represented the sample; additionally, participants had average educational years in school of 17.9 ± 3.5 years. Participant’s biometric information included: weight of 84.1 ± 19.9 kilograms, height of 165.9 ± 18.4 centimeters, and body mass index (BMI) of 30.1 ± 5.2 kg/m^2^. Additionally, there were no significant vascular (blood pressure, cholesterol, fasting glucose, obesity) or genetic (ApoE) differences between clusters.

### Physical function index clusters

Based on the observed changes of the agglomeration schedule and validation, a 3-cluster solution could best discriminate between measures of physical function index and produce satisfactory division of individuals between clusters and was, therefore, selected for the subsequent analysis. Cluster 1 (C1; *n* = 29) is characterized by high overall strength, power, faster dual-task walking time, and higher aerobic capacity. Cluster 3 (C3; *n* = 112) is described as the lowest strength, lower and slowest power output, slower dual task times, and lowest aerobic capacity. Cluster 2 (C3; *n* = 74) is in-between clusters 1 and 3 for all values (Table 2). Significant differences were identified in age and sex between groups. The observed power for significant cognitive variables ranged from 0.65–0.93. Validation for the number of clusters selected and table with cluster centroid distances is in the Supplementary material.

**Table 2:** Cluster centers, demographic, clinical data.

	Cluster1 (*n* = 29)	Cluster 2 (*n* = 74)	Cluster 3 (*n* = 113)	*p-*value
Demographics				
Age	56.9 ± 8.2	60.5 ± 8.0	64.2 ± 7.6	<0.001
Sex (female%)	37.9	66.2	86.7	<0.001
Education	18.1 ± 2.8	17.9 ± 3.3	17.8 ± 4.2	0.922
Anthropometrics and genetics				
Weight	96.9 ± 23.2	89.7 ± 17.5	78.6 ± 14.6	0.009
Height	174.7 ± 9.1	170.3 ± 9.0	163.8 ± 7.4	0.002
BMI	31.5 ± 5.8	30.9 ± 5.7	29.1 ± 4.6	0.090
ApoE (*e4* Positive %)	18.5%	30.9%	22.5%	0.272
Clinical characteristics				
ANU-ADRI Total	2.1 ± 1.5	−1.8 ± 0.8	−2.3 ± 0.7	0.036
ANU-ADRI Protective	−8.9 ± 0.9	−10.3 ± 0.5	−10.7 ± 0.4	0.229
ANU-ADRI Risk	10.9 ± 1.0	8.5 ± 0.6	8.4 ± 0.5	0.078
Resting SBP	126.0 ± 11.4	128.1 ± 10.8	128.6 ± 13.2	0.608
Resting DBP	81.6 ± 9.5	82.3 ± 10.1	82.1 ± 10.4	0.958
Resting HR	73.6 ± 14.5	71.2 ± 12.6	72.6 ± 9.6	0.586
Total cholesterol	199.5 ± 45.6	201.7 ± 41.9	204.9 ± 43.3	0.142
LDL	118.9 ± 35.7	120.9 ± 33.9	122.8 ± 48.2	0.906
HDL	50.4 ± 0.18.2	53.7 ± 17.8	59.4 ± 14.8	0.704
TRG	159.7 ± 121.0	134.1 ± 66.9	136.7 ± 66.2	0.308
Fasting glucose	99.6 ± 14.8	106.5 ± 24.3	100.7 ± 21.7	0.168
Physical function index				
HG right (kg)	37.99	31.66	26.61	< 0.001
HG left (kg)	34.87	30.34	25.23	< 0.001
Average power (W)	659.38	499.22	336.32	< 0.001
Average partial power (W)	548.16	421.54	296.59	< 0.001
Peak power average (W)	1508.42	1020.01	632.97	< 0.001
Average velocity (m/s)	0.68	0.58	0.46	< 0.001
Peak velocity (m/s)	1.16	0.99	0.77	< 0.001
Peak force (*N*)	1504.19	1213.62	933.29	< 0.001
4 m fast (s)	2.16	2.32	2.40	0.022
10 m fast (s)	4.94	5.35	5.63	0.005
4 m DT hab (s)	3.42	3.58	3.81	0.043
4 m DT fast (s)	2.58	2.66	2.93	0.041
10 m DT hab (s)	7.87	8.30	9.11	0.012
10 m DT fast (s)	5.82	6.16	6.81	< 0.001
6MWDT (m)	566.8	546.6	517.6	0.002

Cluster means, HG = hand grip, DT = dual task, hab = habitual, m = meter; SBP = systolic blood pressure; DBP = diastolic blood pressure; HR = heart rate; LDL = low-density lipoprotein; HDL = high-density lipoprotein; TRG = triglyceride; ANU-ADRI = Australian National University-Alzheimer’s disease risk index.

### Cognitive outcomes

#### RBANS

Differences in global cognitive scores (RBANS total score) were found between groups (*F* (2,198) = 3.73, *p* = 0.018; η^2^*_p_ = 0*.036; Table 3), C1 performed significantly better on RBANS total score than C3 (*p* = 0.014). Also, C1 had significantly higher visuospatial scores (*F* (2,197) = 6.28, *p* = 0.003; η^2^*_p_ = 0*.060; Table 3) and Attention Scores than C3 (*F* (2,197) = 3.22, *p* = 0.040; η^2^*_p_ = 0*.032; Table 3). Moreover, Line orientation scores were significantly different between groups (*F* (2,196) = 7.45, *p* = <0.001, η^2^*_p_ = 0*.071; Table 4); C1 had significantly better scores compared to C3 (*p* = 0.004) and C2 performed significantly higher than C3 (*p* = 0.007). Furthermore, significant differences were seen between groups on the figure recall exam, a delayed memory test (*F* (2,196) = 5.45, *p* = 0.005; η^2^*_p_ = 0*.052; Table 4), where C1 performed significantly better than C3 (*p* = 0.011).

**Table 3:** Cluster cognitive domain data.

	Cluster 1 (*n* = 29)	Cluster 2 (*n* = 74)	Cluster 3 (*n* = 113)	*p*-value
Immediate memory	107.1 ± 2.7	100.7 ± 1.5	101.9 ± 1.3	0.109
Visuospatial/constructional	109.9 ± 3.3*	102.7 ± 1.8	97.0 ± 1.5*	0.002
Language	104.4 ± 2.0	103.4 ± 1.1	101.1 ± 0.9	0.201
Attention	116.5 ± 2.8*	111.1 ± 1.6	108.3 ± 1.3*	0.042
Delayed memory	108.1 ± 10.1	114.3 ± 5.6	101.9 ± 4.7	0.257
Total score	111.9 ± 2.1*	104.9 ± 1.5	102.8 ± 1.3*	0.026

Mean ± SE. *indicates significant differences between clusters.

**Table 4:** Cluster individual exam cognitive data.

	Cluster 1 (*n* = 29)	Cluster 2 (*n* = 74)	Cluster 3 (*n* = 113)	*p*-value
Listen learning (IM)	29.6 ± 0.9	28.1 ± 0.5	27.8 ± 0.4	0.337
Story memory (IM)	19.0 ± 0.7	17.6 ± 0.4	18.0 ± 0.3	0.189
Figure copy (V/C)	18.8 ± 0.4	18.1 ± 0.2	17.9 ± 0.2	0.217
Line orientation (V/C)	18.0 ± 0.6^a^	17.0 ± 0.3^b^	15.7 ± 0.3^a,b^	<0.001
Semantic fluency (LAN)	23.4 ± 0.9	22.5 ± 0.5	21.7 ± 0.4	0.206
Picture naming (LAN)	9.7 ± 0.1	9.8 ± 0.1	9.6 ± 0.1	0.264
Coding (ATT)	52.4 ± 1.8	49.5 ± 1.0	48.3 ± 0.8	0.151
Digit span (ATT)	12.9 ± 0.5	12.2 ± 0.3	11.7 ± 0.2	0.132
List RECALL (DM)	6.7 ± 0.4	6.7 ± 0.2	6.5 ± 0.2	0.808
Story recall (DM)	10.1 ± 0.4	9.6 ± 0.2	9.5 ± 0.2	0.430
Figure recall (DM)	15.9 ± 0.7*	14.1 ± 0.4	13.2 ± 0.4*	0.005
List recognition (DM)	19.5 ± 0.2	19.3 ± 0.1	19.5 ± 0.1	0.376
Arrow Match (attention)	36.9 ± 5.1	31.6 ± 3.1	32.2 ± 2.6	0.658
Path Points (Exec Func)	75.6 ± 6.2^a^	66.9 ± 3.5^b^	53.8 ± 2.9^a,b^	0.002
Light Reaction (Inhibition)	32.1 ± 5.3	29.3 ± 3.1	23.2 ± 2.7	0.207
Symbol Match(Processing sp.)	44.6 ± 5.9	39.7 ± 3.4	35.2 ± 2.9	0.337
Item Price (As. Learning)	63.5 ± 5.2^*^	54.6 ± 3.1	47.2 ± 2.7^*^	0.031
Paired imaging (As. Memory)	45.5 ± 7.2	43.2 ± 4.4	45.6 ± 3.0	0.897

Mean ± SE, IM = Immediate memory, V/C = visuospatial/constructional, LAN = Language, ATT = Attention DM = delayed memory, Exec Func = executive functioning, processing sp. = processing speed, As. = associative. *,a,b = indicate significant differences between groups from Bonferroni posthoc analysis.

#### Digital cognitive battery

Among the digital cognitive tasks, there was a difference in path points (executive function) scores between groups (*F* (2,189) = 6.70, *p* = 0.002; η^2^*_p_ = 0*.07; Table 4). C1 demonstrated better executive functioning than C3 (*p* = 0.006), C2 also had significantly higher scores than C3 (*p* = 0.016). Moreover, significant differences were identified between C1 and C3 Item Price (associative learning) scores (*F* (2,184) = 3.53, *p* = 0.044; η^2^*_p_ = 0*.04; Table 4). No other significant differences were found between each cluster during the digital cognitive task.

## Discussion

The purpose of this investigation was to examine if high-risk individuals with a higher physical function index would have better cognitive scores than individuals with a lower physical function index. To our knowledge, this is the first study to examine cognitive differences based on physical function variables grouped together between high-risk individuals. Our *a priori* hypothesis predicted high-risk individuals with higher physical function indexes would display higher cognitive domain scores compared to high-risk individuals with lower physical function indexes. This hypothesis was supported; individuals with a higher physical function index exhibited better global cognitive scores and other cognitive domains than the individuals with a lower physical function index. More specifically, visuospatial domain scores, attention tests, and delayed memory recall performance were significantly better for individuals in cluster 1 compared to individuals in cluster 3. Additionally, individuals in clusters 1 and 2 exhibited better scores on executive functioning and associative learning digital cognitive tasks than individuals in cluster 3. This study highlights the importance of early cognitive examination and tracking brain health through non-invasive measures. Understanding when to implement mitigation measures to combat cognitive decline such as non-pharmaceutical intervention could help alleviate the burden ADRD has on the healthcare system. Non-pharmaceutical interventions could improve physical function index, described in this manuscript, delaying cognitive impairment in high-risk individuals.

This exploratory analysis revealed individuals with a higher physical function indexing (cluster 1) had better global cognitive scores than individuals with a lower physical function index (cluster 3). Physical activity and exercise studies have shown men and women who are aerobically active in mid and late life are at lower risk for global cognitive decline (4, 14, 41, 42). Although some of these investigations were longitudinal, researchers found higher step counts and average metabolic equivalents have greater cognitive benefits and neuroprotection cross-sectionally. Moreover, our study showed executive functioning and delayed memory tasks were greater for individuals in cluster 1 compared to individuals in cluster 3. The Doetinchem Cohort Study exhibited the similar results with 6–11 year follow-up analyses (16). Researchers demonstrated that the intensity and variation of physical activities were positively associated with processing speed, memory, mental flexibility, and overall cognitive function—duration was not associated with better cognitive functioning. Likewise, another cross-sectional study found greater executive functioning among older men and women with higher physical activity and greater energy expenditure rates (12).

Similarly, the current study showed individuals in cluster 1 exhibited 34% greater average muscular strength values than individuals in cluster 3; and, cluster 2 exhibited 18% higher average muscular strength output than cluster 3. Previous investigations indicate higher handgrip values are associated with executive function, attention, memory, and overall cognition, similar to the results observed in this current study (43–45). Moreover, individuals in cluster 1 and 2 displayed 65 and 39% higher average lower body power than cluster 3, respectively. Additionally, cluster 1 exhibited 28% greater average lower body velocity than cluster 3; while cluster 2 on average displayed 23% higher lower body velocity than cluster 3. These current results are similar to previous literature in which higher muscular power and velocity scores had strong relationships with overall cognition and executive function scores (11, 13, 46). To our knowledge, there are no studies examining the relationship between lower body muscular power/velocity, grouped together, and cognition in middle-to-older high-risk adults. Results should be explored further due to the neural component of power and velocity. Additionally, our study showed quicker dual-task velocity and fast walking speeds (cluster 1 and 2) were associated with better cognitive functioning. Our results coincide with previous studies where faster walking velocity on both habitual and fast tasks were associated with better cognitive functioning (7–9).

Due to the cross-sectional nature of our study, we cannot demonstrate the causality of physical function affecting cognitive function. However, various clinical trials are examining the impact of different types of exercise on cognitive function and the physiological mediators between the relationship (5, 10, 11, 47–49). Mechanisms such as increased cerebral blood flow, augmented prefrontal oxygenation, neuronal network flexibility, neurogenesis through brain derived neurotropic factor and IGF-1 are potential mechanisms that may help explain why exercise and increased physical function index improve cognitive function in middle-to-older aged adults (11, 13, 50, 51). Future studies should examine mechanisms to elucidate biological and physiological pathways in which exercise and physical function may impact cognition and brain health.

This current investigation had limitations due to the sample, sample size, clustering results, and effect size. The sample was mainly Caucasian Americans which limits the generalizability of the results. Heterogeneity in different populations may change current results from this study due to education, genetics, and other social determinants of health. Additionally, the test used created imbalances in the weight of the physical function indexes. More gait speed tests are used in the analysis than other physical function variables, muscular fitness variables outnumber aerobic capacity variable as well. Moreover, it should be noted clusters have imbalances due age, sex, weight, and height allowing for those with more body mass and height to have greater mechanical advantages in muscular strength/power tests and walking distance test. However, it should be noted that age and sex was controlled for in each statistical analysis, while there were no significant differences in BMI between clusters. Additionally, this study sample size and effect size of results, which is small to medium, hindered the conclusions that could be made from the physical function indexes. A more robust sample size may help examine each variable on cognition. Lastly, cross-sectional data were used to evaluate these current findings, which limits the conclusions that can be made as it only examines a single time point.

This is the first study examining cognitive differences between combined physical function variables in high-risk individuals. Results suggest evaluating cognition through physical function variables combined may provide a more efficient way of evaluating physical function on cognition. Cluster 1 surpassed the minimal clinically important difference, for RBANS, of >3.3 against both cluster 2 and cluster 3 (52). This indicates the value in combining physical function variables into an index instead of examining a singular domain of physical function. More research is needed on a larger scale, incorporating genetics, to determine if higher functioning cluster values can offset genetic risk variants. Additional research is warranted to also understand cut-off values for normal cognitive function individuals without high-risk for cognitive decline and individuals with mild cognitive decline based on age. Furthermore, examining differences in early blood-based AD biomarker and brain imaging data would provide more support for this method. This is a prospective method of evaluating cognition that is less invasive and cost-efficient. Longitudinal research is required to foster well-rounded generalizable results, which neurologists could apply to cognitive decline evaluation.

In conclusion, the results of this current study showed value in evaluating individual’s cognitive outcomes through physical function indexes. Participant’s physical function index output delineated differences in global cognition, spatial ability, delayed memory, associative learning, and executive functioning scores. Future studies should examine physical function index clustering over time and between cognitive normal and mild cognitive impaired patients. Overall, the goal is to detect early cognitive decline through widely accessible measures and to implement timely mitigation measures to improve overall health and maintain brain health.

## Data Availability

The raw data supporting the conclusions of this article will be made available by the authors, without undue reservation.
