# Supplementary material for: The examination of physical function and cognitive outcomes in middle-to-older high-risk adults: an unsupervised clustering method
Source: Front Public Health. 2025 May 20;13:1351658. doi: 10.3389/fpubh.2025.1351658 (PMC12132019; doi:10.3389/fpubh.2025.1351658)

**Supplementary Material**

**Internal Validation**

| **Connectivity** | **2.9290** | **hierarchical** | **2** |
| --- | --- | --- | --- |
| **Dunn** | **0.5425** | **hierarchical** | **3** |
| **Silhouette** | **0.5177** | **hierarchical** | **2** |

**Our selection was based on the maximum number 3 per Dunn score.**

**
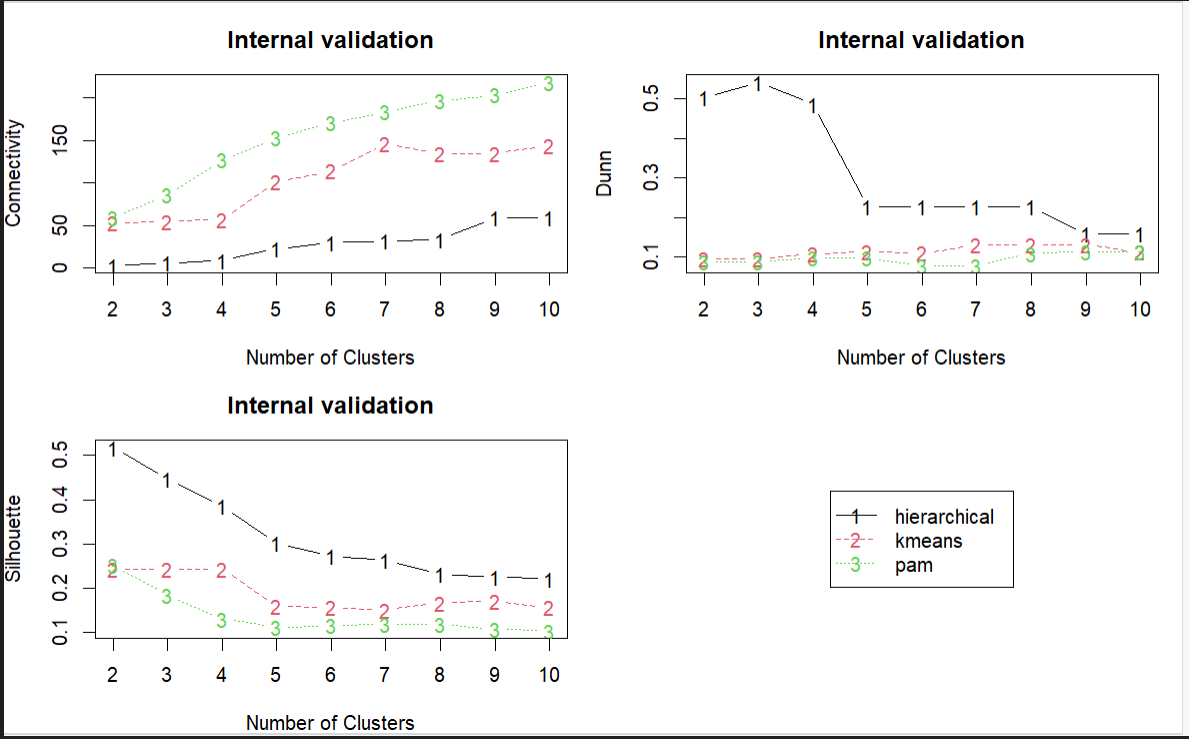
**

**Therefore, using 3 clusters is the appropriate selection for our current analysis.**

*Table 1 supp. Distance between Cluster Centroids*


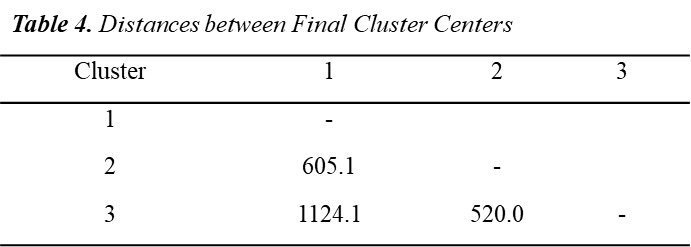

Supplement: Supplementary file 1 [file Supplementary_file_1.docx]
